# A Glutamatergic Medial Prefrontal Cortex–Locus Coeruleus Circuit Drives Intestinal Dysmotility in Diarrhea-Predominant Irritable Bowel Syndrome

**DOI:** 10.3390/ijms27020681

**Published:** 2026-01-09

**Authors:** Shu-Man Jia, Kai-Qi Wang, Shu-Fen Hu, Rui-Xia Weng, Kun Liu, Qian Sun, Rui Li

**Affiliations:** 1 The First Affiliated Hospital of Soochow University, Suzhou 215006, China; jiashuman11@163.com (S.-M.J.); w18862396273@163.com (K.-Q.W.); wengruixia@suda.edu.cn (R.-X.W.); 2 Jiangsu Key Laboratory of Neuropsychiatric Diseases and Institute of Neuroscience, Soochow University, Suzhou 215123, China; sfhu@suda.edu.cn (S.-F.H.); lkun_0808@126.com (K.L.)

**Keywords:** diarrhea-predominant irritable bowel syndrome, intestinal dysmotility, medial prefrontal cortex, glutamatergic neuron, locus coeruleus

## Abstract

Diarrhea-predominant irritable bowel syndrome (IBS-D) is a common chronic disorder of gut–brain interaction characterized by intestinal dysmotility. Central sensitization has a proposed role in intestinal dysmotility, yet the precise neural circuits and mechanisms remain poorly understood. In this study, we established a neonatal maternal deprivation plus restraint stress (NMD + RS) mouse model that recapitulates key diarrhea-like phenotypes. Neural activation mapping revealed a significant upregulation of c-Fos expression within the medial prefrontal cortex (mPFC) and locus coeruleus (LC), which was predominantly localized to glutamatergic neurons. Chemogenetic inhibition of mPFC glutamatergic neurons suppressed intestinal dysmotility, whereas the activation of mPFC glutamatergic neurons evoked intestinal dysmotility in control mice. Furthermore, viral tracing revealed direct projections from mPFC neurons to glutamatergic neurons in the LC. Subsequent chemogenetic manipulation of these LC glutamatergic neurons receiving projection from mPFC neurons similarly regulated intestinal motility, demonstrating a functional downstream node. Critically, selective activation of the mPFC-LC glutamatergic circuit significantly induced intestinal dysmotility in CON mice. In contrast, inhibition of the mPFC-LC glutamatergic circuit significantly ameliorated intestinal dysmotility in NMD + RS mice. Our findings proved that the enhanced activity of the mPFC-LC circuit led to intestinal dysmotility in NMD + RS mice, hopefully providing new mechanistic perspectives and a potential neuromodulatory target for clinical management of IBS.

## 1. Introduction

Irritable bowel syndrome (IBS) is a prevalent chronic functional gastrointestinal disorder characterized by recurrent abdominal pain and altered bowel habits [1]. According to the Rome IV criteria, diarrhea-predominant IBS (IBS-D) is the most prevalent among the four subtypes and comprises about one-third of IBS patients [2,3,4]. The etiology and pathological mechanisms of IBS-D are complex. While prior studies have mainly focused on peripheral factors—including gut microbiota imbalance, weakened intestinal barrier function, and mild inflammation—the central mechanisms remain largely elusive. Current therapies targeting these peripheral pathways have demonstrated limited clinical efficacy. Therefore, further investigation into the central mechanisms of IBS-D and the development of effective therapeutics are essential to advance clinical management and improve patients’ quality of life.

Emerging evidence indicates that the central nervous system is a key driver of IBS-D pathophysiology via the brain–gut axis [5,6]. Functional magnetic resonance imaging (fMRI) studies have shown both functional and structural alterations in several brain regions of IBS-D patients, including the medial prefrontal cortex (mPFC), locus coeruleus, insula, and striatum, among others. The mPFC is regarded as a key “visceromotor” area that integrates visceral sensory and motor information [7,8,9] and exerts top-down control over gastrointestinal function through the brain–gut axis, supporting its pivotal role in IBS-D-related intestinal dysmotility.

The locus coeruleus (LC) has long been recognized as a central noradrenergic hub, playing a key role in regulating arousal, attention, and nociception [10,11,12]. However, recent studies highlighted the specific involvement of LC glutamatergic neurons, which were activated by stress to modulate stress perception, emotional regulation, and behavioral adaptation [13,14]. Anatomical and functional studies have demonstrated that the mPFC directly projects to the LC, influencing emotional processing and stress regulation [15,16,17,18]. The mPFC-LC circuit may be critically involved in the top-down dysregulation of intestinal motility characteristic of IBS-D.

In this study, we proposed the hypothesis that the enhanced glutamatergic neuron activity in the mPFC region activated the LC glutamatergic neurons, thereby leading to diarrhea in IBS-D. Previous studies have reported that early-life stress or adverse stimuli are important contributing factors to IBS-D [19,20]. To test this hypothesis, we employed the neonatal maternal deprivation plus restraint stress (NMD + RS) model—an established paradigm for simulating IBS-D—to induce diarrhea-like symptoms in mice [21,22,23,24,25]. To test this hypothesis, the neonatal maternal deprivation combined with restraint stress (NMD + RS) model was adopted to induce diarrhea in mice [24,25]. This study identified that the regulation of the mPFC-LC circuit could alter intestinal motility behaviors, hoping to provide a new target for clinical central neuromodulatory therapies (such as transcranial magnetic stimulation) in IBS-D.

## 2. Results

### 2.1. NMD + RS Mice Exhibited Intestinal Dysmotility Behaviors

**Figure 1:**
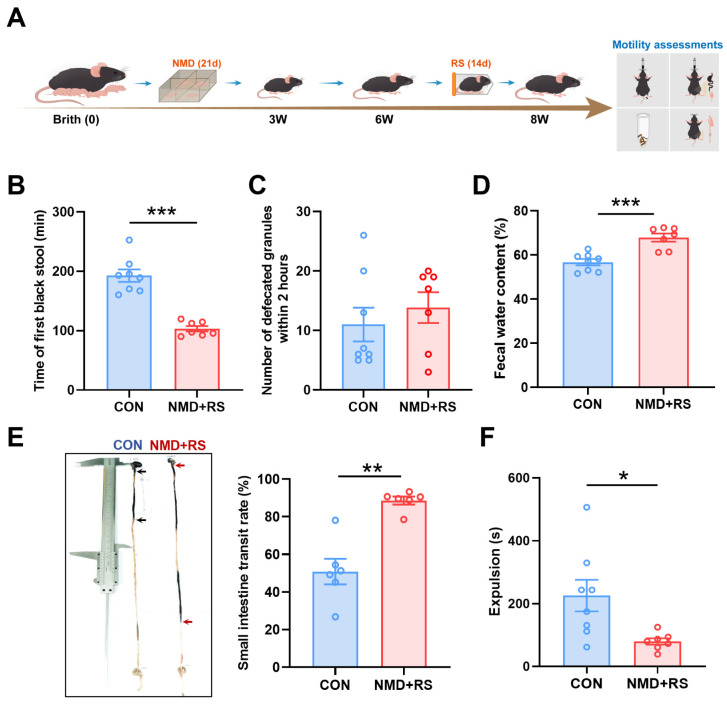
NMD + RS mice exhibited intestinal dysmotility behaviors. (**A**) The flowchart of the NMD + RS model construction and assessments of intestinal motility. (**B**) Statistical chart of first black stool time in CON group (*n* = 8) and NMD + RS group (*n* = 7), unpaired *t* test, *** *p* < 0.001. (**C**) The number of defecated granules within 2 h was counted in the CON group (*n* = 8) and the NMD + RS group (*n* = 7), Mann–Whitney U test, *p* = 0.5939. (**D**) Statistical chart of fecal water content in the CON group (*n* = 8) and NMD + RS group (*n* = 7), unpaired *t* test, *** *p* < 0.001. (**E**) Representative images of small intestine transit rate (**left**) and statistical chart of small intestine transit rate (**right**) in the CON group and NMD + RS group. Black arrows represented CON group, red arrows represented NMD + RS group, *n* = 6 per group, Mann–Whitney U test, ** *p* < 0.01. (**F**) Statistical chart of intestine expulsion time in the CON group (*n* = 8) and NMD + RS group (*n* = 7), unpaired *t* test, * *p* < 0.05. Blue bars represented the CON group, and pink bars represented the NMD + RS group. NMD + RS: neonatal maternal deprivation plus restraint stress; CON: control.

The model of neonatal maternal deprivation combined with adult restraint stress (NMD + RS) was established to mimic the intestinal dysmotility in IBS-D [24,25], and a series of intestinal motility assessments was conducted to evaluate the gastrointestinal motility in CON and NMD + RS mice (Figure 1A). Compared to the CON group, NMD + RS mice exhibited a significantly shorter latency to the first black stool (Figure 1B) and an increased fecal water content (Figure 1C), but no significant difference in defecation frequency within 2 h (Figure 1D). Additionally, total intestinal transport time and colonic transit assay were performed to detect the gastrointestinal motility. The NMD + RS group showed an enhanced small-intestinal transit rate (Figure 1E) and a faster glass-bead expulsion time (Figure 1F). These data reveal the successful establishment of the NMD + RS mouse model, which offers an appropriate animal model for subsequent studies on the diarrheal mechanisms of IBS-D.

### 2.2. The Expression of c-Fos Was Increased in the mPFC in NMD + RS Mice

**Figure 2:**
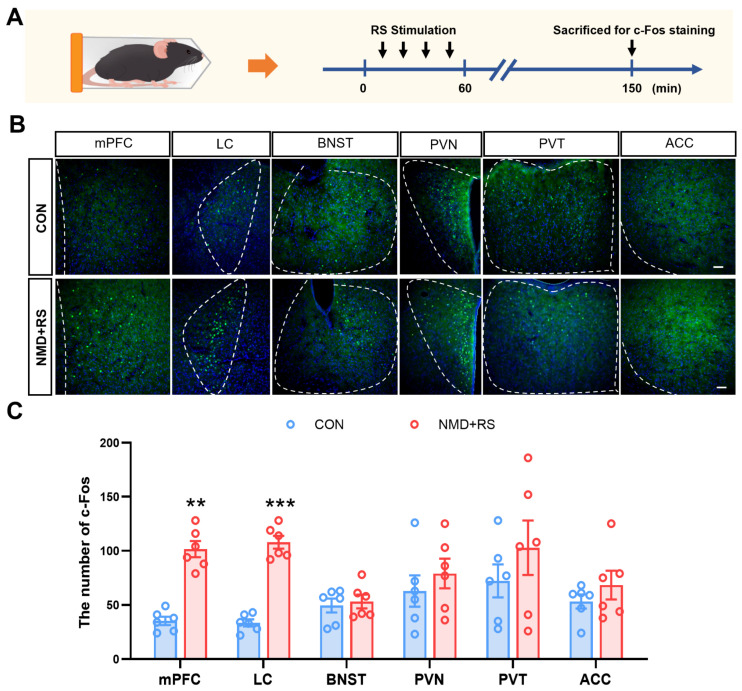
mPFC and LC neurons were involved in intestinal dysmotility in NMD + RS mice. (**A**) Schematic of the RS stimulation protocol. (**B**) Representative images of c-Fos expression in response to RS stimulation across multiple brain regions in CON and NMD + RS mice. The dashed areas indicate the location of each corresponding brain region. (**C**) Quantification of the number of c-Fos^+^ cells in CON and NMD + RS mice (each data point represents a brain slice per region, *n* = 3 mice per group), two-way ANOVA followed by Sidak’s multiple comparisons test, ** *p* < 0.01, *** *p* < 0.001. Scale bars, 50 μm. Blue bars represented the CON group, and pink bars represented the NMD + RS group. NMD + RS: neonatal maternal deprivation plus restraint stress; RS: restraint stress; mPFC: medial prefrontal cortex; LC: locus coeruleus; BNST: bed nucleus of the stria terminalis; PVN: paraventricular nucleus of the hypothalamus; PVT: paraventricular thalamic nucleus; ACC: anterior cingulate cortex.

To systematically identify brain regions regulating intestinal dysmotility in NMD + RS mice, CON and NMD + RS mice were subjected to a 60 min restraint stress, and neural activity was subsequently assessed by whole-brain c-Fos mapping (Figure 2A). Immunofluorescence staining showed that among multiple brain regions related to the intestinal motility, the c-Fos expression in the mPFC and LC regions from NMD + RS mice was higher than from CON mice, whereas no significant differences were observed in the bed nucleus of the stria terminalis (BNST), paraventricular nucleus of the hypothalamus (PVN), paraventricular thalamic nucleus (PVT), and anterior cingulate cortex (ACC) between CON and NMD + RS mice (Figure 2B,C). These data suggest that activation of the mPFC and LC may be associated with the development of diarrhea in IBS-D.

### 2.3. Enhanced Glutamatergic Neuron Activity in the mPFC Modulates Diarrhea Behaviors

**Figure 3:**
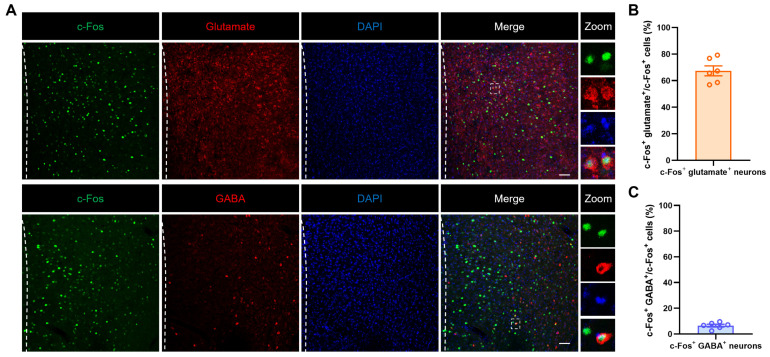
Restraint stimulation primarily activated glutamatergic neurons in the mPFC region. (**A**) Representative images illustrating c-Fos (green) co-labeled with glutamate (red, top) and GABA (red, bottom) in the mPFC. The area to the right of the dashed line indicates the location of the mPFC. The images on the right correspond to an enlarged view of the dashed box. Scale bar, 50 μm. (**B**) Statistical graph of c-Fos co-labeling ratio in glutamatergic neurons. Each data point represents a brain slice, *n* = 3 mice. (**C**) Statistical graph of c-Fos co-labeling ratio in GABA neurons. Each data point represents a brain slice, *n* = 3 mice. mPFC: medial prefrontal cortex. GABA: gamma-aminobutyric acid.

To verify the types of mPFC neurons activated by restraint stress stimulation, immunofluorescence staining was performed. The results showed that over 67.39% of c-Fos positive cells co-localized with glutamate (marker for glutamatergic neurons), while only 6.49% were co-labeled with GABA (marker for GABAergic neurons) (Figure 3A–C). This data suggested that the mPFC glutamatergic neurons may be involved in diarrhea in NMD + RS mice.

**Figure 4:**
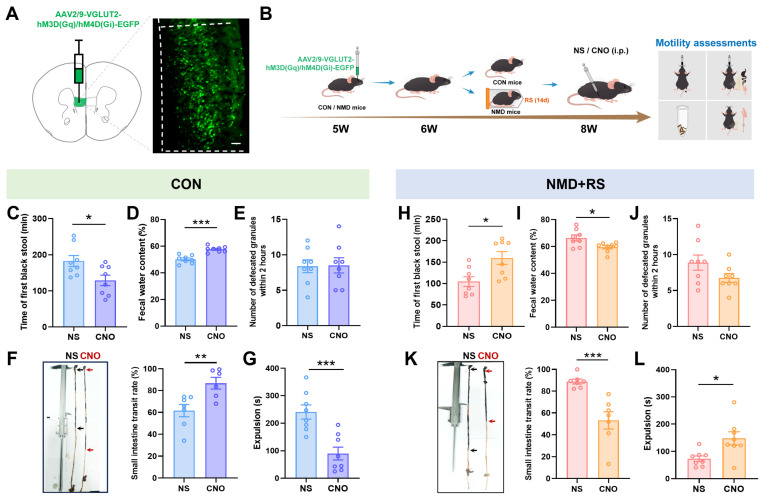
mPFC ^Glu^ neurons were involved in regulating diarrhea in NMD + RS mice. (**A**) Illustration of the strategy of chemogenetic viral vector injection and the fluorescence image of viral expression in the mPFC region. The dashed areas indicate the location of mPFC region. Scale bar, 50 μm. (**B**) Experimental flowchart and assessments of intestinal motility protocol. (**C**) The first black stool time was measured between CON mice receiving NS groups and CNO groups, *n* = 8 per group, unpaired *t* test, * *p* < 0.05. (**D**) Fecal water content was detected between CON mice receiving NS groups and CNO groups, *n* = 8 per group, unpaired *t* test, *** *p* < 0.001. (**E**) The number of defecated granules within 2 h was counted between CON mice receiving NS groups and CNO groups, *n* = 8 per group, unpaired *t* test, *p* = 0.9302. (**F**) Representative images of small intestine transit rate (**left**) and statistical chart of small intestine transit rate (**right**) between CON mice receiving NS groups (*n* = 7) and CNO groups (*n* = 6), black arrows represented CON mice receiving NS group, red arrows represented CON mice receiving CNO group, unpaired *t* test, ** *p* < 0.01. (**G**) The time to intestine expulsion was measured between CON mice receiving NS groups and CNO groups, *n* = 8 per group, unpaired *t* test, *** *p* < 0.001. (**H**) In NMD + RS mice, the first black stool time was measured between NS groups and CNO groups, *n* = 8 per group, unpaired *t* test, * *p* < 0.05. (**I**) In NMD + RS mice, fecal water content was detected in NS groups and CNO groups, *n* = 8 per group, unpaired *t* test, * *p* < 0.05. (**J**) In NMD + RS mice, the number of defecated granules within 2 h was counted in NS groups and CNO groups, *n* = 8 per group, unpaired *t* test, *p* = 0.1054. (**K**) Representative images of small intestine transit rate (**left**) and statistical chart of small intestine transit rate (**right**) in NMD + RS mice receiving NS and CNO groups, black arrows represented NMD + RS mice receiving NS group, red arrows represented NMD + RS mice receiving CNO group, *n* = 7 per group, unpaired *t* test, *** *p* < 0.001. (**L**) The time to intestine expulsion was measured in NMD + RS mice receiving NS and CNO groups, *n* = 8 per group, unpaired *t* test, * *p* < 0.05. Blue bars represented CON mice receiving NS group, purple bars represented CON mice receiving CNO group, pink bars represented NMD + RS mice receiving NS group, yellow bars represented NMD + RS mice receiving CNO group. NMD + RS: neonatal maternal deprivation plus restraint stress; mPFC: medial prefrontal cortex; NS: normal saline; CNO: clozapine-N-oxide.

To further investigate the regulatory role of mPFC hyperexcitability in intestinal motility, the chemogenetic virus (AAV2/9-VGLUT2 hM3D (Gq)/hM4D (Gi)-EGFP) was microinjected into the mPFC at 5 W (Figure 4A). After a one-week recovery period, mice underwent two weeks of restraint stress. Neuronal activation hM3D (Gq) or inhibition hM4D (Gi) was induced by intraperitoneal injection of clozapine-N-oxide (CNO) or normal saline (NS) 45 min prior to behavioral assessments (Figure 4B). In CON mice, CNO administration significantly shortened the time to first black stool (Figure 4C), increased fecal water content (Figure 4D), enhanced the small-intestinal transit rate (Figure 4F), and accelerated glass-bead expulsion (Figure 4G), although no significant change was observed in 2 h defecation frequency (Figure 4E). In NMD + RS mice, CNO treatment markedly prolonged the time to first black stool (Figure 4H), decreased fecal water content (Figure 4I), reduced the small-intestinal transit rate (Figure 4K), and delayed glass-bead expulsion (Figure 4L), whereas no significant alteration in 2 h defecation frequency (Figure 4J). These data indicated that the chemogenetic modulation of glutamatergic neuronal activity in the mPFC mediated intestinal motility and contributed to diarrhea-like symptoms in IBS-D.

### 2.4. Restraint Stimulation Primarily Activated Glutamatergic Neurons in the LC Region

**Figure 5:**
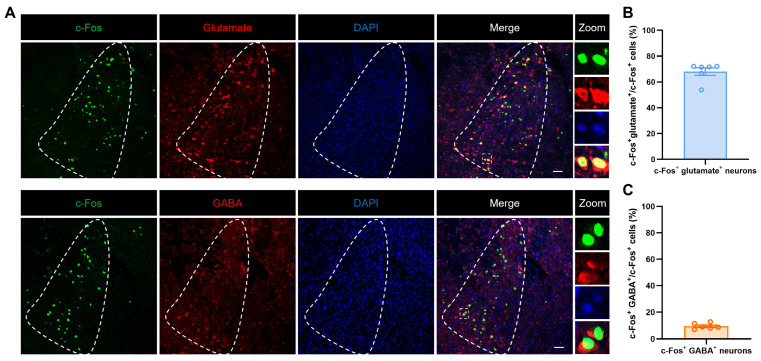
Restraint stimulation mainly activated glutamatergic neurons in the LC region. (**A**) Representative images illustrating c-Fos (green) co-labeled with glutamate (red, top) and GABA (red, bottom) in the LC region. The dashed areas indicate the location of LC region. The images on the right correspond to an enlarged view of the dashed box. Scale bar, 50 μm. (**B**) Statistical graph of c-Fos co-labeling ratio in glutamatergic neurons. Each data point represents a brain slice, *n* = 3 mice. (**C**) Statistical graph of c-Fos co-labeling ratio in GABA neurons. Each data point represents a brain slice, *n* = 3 mice. LC: locus coeruleus; GABA: gamma-aminobutyric acid.

To verify the types of LC neurons activated by restraint stress stimulation, immunofluorescence staining was performed. The results showed that over 67.97% of c-Fos positive cells co-localized with glutamate (marker for glutamatergic neurons), while only 9.52% were co-labeled with GABA (marker for GABAergic neurons) (Figure 5A–C). This data suggested that the LC glutamatergic neurons may be involved in diarrhea in NMD + RS mice.

### 2.5. mPFC ^Glu^ Neurons Projected to the LC Region

**Figure 6:**
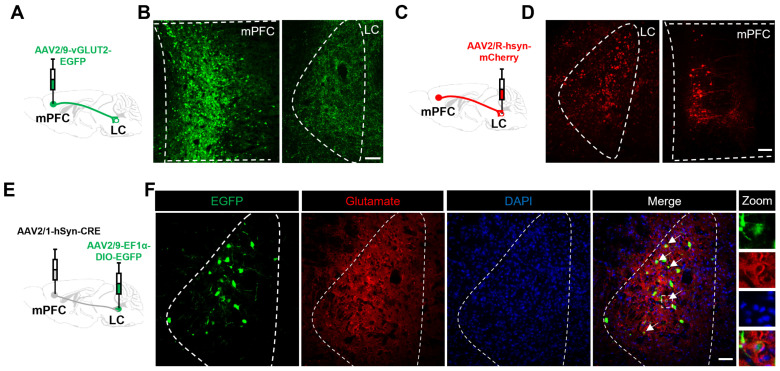
mPFC^Glu^ neurons projected to the LC region. (**A**) Pattern diagram of the anterograde viral tracing strategy in the mPFC region. (**B**) Images of viral expression in the mPFC glutamatergic neurons (**left**) and their axon terminals in the LC (**right**), *n* = 3. (**C**) Pattern diagram of the retrograde virus tracing strategy in the LC region. (**D**) Images of viral expression at the injection site (terminals in LC, **left**) and in the retrogradely labeled neurons (somata in mPFC, **right**), *n* = 3. (**E**) Schematic of monosynaptic anterograde tracing in the mPFC and LC region. (**F**) Representative image illustrating co-expression of EGFP-labeled fluorescent signal and Glutamate (red) in the LC. Scale bar, 50 μm (*n* = 3 mice). The dashed areas indicate the location of each corresponding brain region. mPFC: medial prefrontal cortex; LC: locus coeruleus.

To investigate the LC glutamatergic neurons involved in mPFC hyperexcitability in diarrhea in NMD + RS mice, circuit viral tracing techniques were conducted. The anterograde tracer virus (AAV2/9-vGLUT2-EGFP) was microinjected into the mPFC (Figure 6A), and a large number of axon terminals were observed in the LC region (Figure 6B). It suggests that the glutamatergic neurons in the mPFC may anterograde project to the LC region. Subsequently, the retrograde tracer virus (AAV2/R-hSyn-mCherry) was microinjected into the LC region (Figure 6C), and the apparent mCherry signal was detected in the mPFC region (Figure 6D), which demonstrates that the mPFC neurons exhibited anterograde projection to the LC region. To investigate whether there is a direct projection from mPFC glutamatergic neurons to the LC, a Cre-DIO-dependent dual-virus model that selectively expressed EGFP was used in a projection-specific manner. A monosynaptic anterograde virus (AAV2/1-hSyn-Cre) was microinjected into mPFC, and a locally expressed virus (AAV2/9-EF1α-DIO-EGFP) was microinjected into LC (Figure 6E). A large number of EGFP signals were obviously detected in the LC region. Immunofluorescence staining further showed that the EGFP signal in the LC region was mainly co-expressed with glutamate (Figure 6F), which further supported the notion that mPFC glutamatergic neurons exhibited an anterograde projection to LC glutamatergic neurons.

### 2.6. LC ^mPFC-Glu^ Were Involved in the Regulation of Diarrhea Behaviors

**Figure 7:**
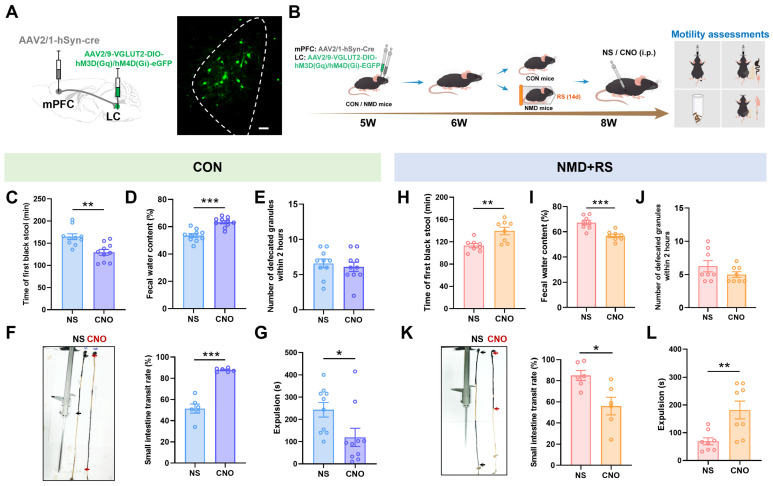
LC ^mPFC → Glu^ neurons were involved in regulating diarrhea in NMD + RS mice. (**A**) Pattern diagram of chemogenetic viral vector microinjection (**left**) and a representative fluorescence image showing viral expression in the LC region (**right**, the dashed areas). Scale bar, 50 μm. (**B**) Experimental flowchart and assessments of intestinal motility protocol. (**C**) In the CON group, the first black stool time was measured in the NS and CNO groups, *n* = 10 per group, Mann–Whitney U test, ** *p* < 0.01. (**D**) Fecal water content was detected in NS and CNO groups, *n* = 10 per group, unpaired *t* test, *** *p* < 0.001. (**E**) The number of defecated granules within 2 h of collection was counted between the NS groups and CNO groups, *n* = 10 per group, unpaired *t* test, *p* = 0.5864. (**F**) Representative images of small intestine transit rate (**left**) and statistical chart of small intestine transit rate (**right**) between NS groups and CNO groups, black arrows represented CON mice receiving NS group, red arrows represented CON mice receiving CNO group, *n* = 6 per group, unpaired *t* test, *** *p* < 0.001. (**G**) The time to intestine expulsion was measured in NS and CNO groups, *n* = 10 per group, Mann–Whitney U test, * *p* < 0.05. (**H**) In the NMD + RS model, the first black stool time was measured after receiving NS groups and CNO groups, *n* = 8 per group, unpaired *t* test, ** *p* < 0.01. (**I**) Fecal water content was detected in NS and CNO groups, *n* = 8 per group, unpaired *t* test, *** *p* < 0.001. (**J**) The number of defecated granules within 2 h was counted in NS groups and CNO groups, *n* = 8 per group, Mann–Whitney U test, *p* = 0.3313. (**K**) Schematic diagram of small intestine transit rate (**left**) and statistical chart of small intestine transit rate (**right**) in receiving NS and CNO groups, black arrows represented NMD + RS mice receiving NS group, red arrows represented NMD + RS mice receiving CNO group, *n* = 6 per group, unpaired *t* test, * *p* < 0.05. (**L**) The time to intestine expulsion was measured from NS and CNO groups, *n* = 8 per group, unpaired *t* test, ** *p* < 0.01. Blue bars represented CON mice receiving NS group, purple bars represented CON mice receiving CNO group, pink bars represented NMD + RS mice receiving NS group, yellow bars represented NMD + RS mice receiving CNO group. NMD + RS: neonatal maternal deprivation plus restraint stress; mPFC: medial prefrontal cortex; LC: locus coeruleus; NS: normal saline; CNO: clozapine-N-oxide.

To further explore the regulatory role of LC glutamatergic neurons receiving projections from mPFC neurons (LC ^mPFC → Glu^) in intestinal motility, a Cre-DIO-dependent chemogenetic dual-virus model was conducted in the CON and NMD + RS mice. A monosynaptic anterograde virus (AAV2/1-hSyn-Cre) was injected into the mPFC region, and chemogenetic viruses (AAV2/9-VGLUT2-DIO-hM3D (Gq)-EGFP/AAV2/9-VGLUT2-DIO-hM4D (Gi)-EGFP) were microinjected into the LC at 5W (Figure 7A). Then the NMD + RS mice were given a one-week rest period, followed by two weeks of restraint stress. Neurons were activated by hM3D (Gq) or inhibited by hM4D (Gi) via intraperitoneal injection of clozapine-N-oxide (CNO) or normal saline (NS) 45 min prior to testing (Figure 7B). In control (CON) mice, chemogenetic activation of LC ^mPFC → Glu^ neurons reduced the time to first black stool (Figure 7C), elevated fecal water content (Figure 7D), increased small-intestinal transit rate (Figure 7F), and accelerated glass-bead expulsion (Figure 7G), but there was no significant difference in defecation frequency within 2 h (Figure 7E). In NMD + RS mice, chemogenetic inhibition of LC ^mPFC → Glu^ neurons significantly delayed time to first black stool (Figure 7H), decreased fecal water content (Figure 7I), reduced small intestinal propulsion rate (Figure 7K), and increased glass-bead expulsion time (Figure 7L) in NMD model mice treated with CNO, whereas no significant difference in defecation frequency within 2 h (Figure 7J). These data indicated that the regulation of the LC ^mPFC → Glu^ neurons’ activity mediated intestinal dysmotility in IBS-D.

### 2.7. The mPFC-LC Glutamatergic Circuit Regulated Diarrhea Behaviors

**Figure 8:**
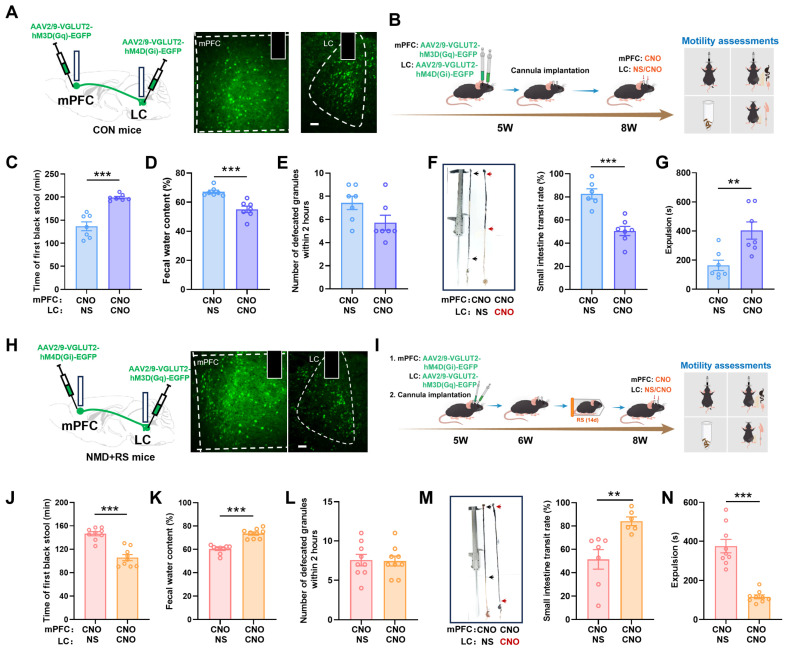
mPFC^Glu^-LC^Glu^ neural circuits regulated diarrhea in mice. (**A**) Schematic diagram of chemogenetic virus injection and cannula implantation in mPFC and LC (**left**) and a representative fluorescence image showing viral expression in the mPFC and LC regions (**right**, the dashed areas) in CON mice. Scale bar, 50 μm. (**B**) Experimental flowchart and assessments of intestinal motility protocol. (**C**) In CON mice, the first black stool time was measured in mPFC receiving CNO and LC receiving NS groups and in two regions receiving g CNO groups, *n* = 7 per group, unpaired *t* test, *** *p* < 0.001. (**D**) Fecal water content was detected in two groups, *n* = 7 per group, Mann–Whitney U test, *** *p* < 0.001. (**E**) The number of defecated granules within 2 h was counted in two groups, *n* = 7 per group, unpaired *t* test, *p* = 0.0698. (**F**) Schematic diagram of small intestine transit rate (**left**) and statistical chart of small intestine transit rate (**right**) in receiving NS groups (*n* = 6) and receiving CNO groups (*n* = 7), black arrows represented CON (CNO in mPFC + NS in LC) group, red arrows represented CON (CNO in mPFC + CNO in LC) group, unpaired *t* test, *** *p* < 0.001. (**G**) The intestine expulsion time was measured in two groups, *n* = 7 per group, unpaired *t* test, ** *p* < 0.01. (**H**) In NMD + RS mice, a diagram of chemogenetic virus injection and cannula implantation in mPFC and LC (**left**) and a representative fluorescence image showing virus expression in the mPFC and LC regions (**right**, the dashed areas). Scale bar, 50 μm. (**I**) Experimental flowchart and assessments of intestinal motility protocol. (**J**) In NMD + RS mice, the first black stool time was measured in the mPFC receiving CNO and LC receiving NS groups and receiving CNO groups, *n* = 9 per group, unpaired *t* test, *** *p* < 0.001. (**K**) Fecal water content was detected in two groups, *n* = 9 per group, Mann–Whitney U test, *** *p* < 0.001. (**L**) The number of defecated granules within 2 h was counted in two groups, *n* = 9 per group, Mann–Whitney U test, *p* = 0.9767. (**M**) Schematic diagram of small intestine transit rate (**left**) and statistical chart of small intestine transit rate (**right**) in receiving NS groups (*n* = 7) and CNO groups (*n* = 6), black arrows represented NMD + RS (CNO in mPFC + NS in LC) group, red arrows represented NMD + RS (CNO in mPFC + CNO in LC) group, unpaired *t* test, ** *p* < 0.01. (**N**) The time to intestine expulsion was measured from two groups, *n* = 9 per group, unpaired *t* test, *** *p* < 0.001. Blue bars represented CON (CNO in mPFC + NS in LC) group, purple bars represented CON (CNO in mPFC + CNO in LC) group, pink bars represented NMD + RS (CNO in mPFC + NS in LC) group, and yellow bars represented NMD + RS (CNO in mPFC + CNO in LC) group. NMD + RS: neonatal maternal deprivation plus restraint stress; mPFC: medial prefrontal cortex; LC: locus coeruleus; NS: normal saline; CNO: clozapine-N-oxide.

Our previous data proved that glutamatergic neurons in the mPFC and LC regions mediated intestinal dysmotility behaviors and that mPFC regions exerted an anterograde projection of glutamatergic neurons to the LC region. However, the function of the mPFC-LC glutamatergic circuit in intestinal dysmotility remains unclear. To further investigate the role of the mPFC-LC glutamatergic circuit, we employed a dual-chemogenetic approach to bidirectionally control the activity of this pathway. In control (CON) mice, we injected AAV2/9-VGLUT2-hM3D (Gq)-EGFP (activator) into the mPFC and AAV2/9-VGLUT2-hM4D (Gi)-EGFP (inhibitor) into the LC (Figure 8A,B). Compared to control mice receiving CNO in the mPFC and saline (NS) in the LC via intracerebral cannulas, the simultaneous chemogenetic activation of mPFC glutamatergic neurons and inhibition of LC glutamatergic neurons significantly delayed the time to first black stool, decreased fecal water content, reduced the small intestinal propulsion rate, and prolonged glass-bead expulsion time (Figure 8C–G).

Conversely, in NMD + RS model mice, we applied the opposite strategy: AAV2/9-VGLUT2-hM4D (Gi)-EGFP (inhibitor) was injected into the mPFC and AAV2/9-VGLUT2-hM3D (Gq)-EGFP (activator) into the LC (Figure 8H, I). After the stress paradigm, compared to the control (CNO in mPFC + NS in LC) group, simultaneous inhibition of mPFC glutamatergic neurons and activation of LC glutamatergic neurons accelerated the time to first black stool, increased fecal water content, enhanced the small intestinal propulsion rate, and shortened the glass-bead expulsion time (Figure 8J–N). The above results further suggested that the activation of the mPFC-LC glutamatergic circuit was sufficient to promote intestinal dysmotility behaviors in IBS-D.

## 3. Discussion

IBS-D is a highly prevalent clinical disorder featuring a complex and multifactorial pathophysiology. Previous studies primarily concentrated on peripheral mechanisms [26]. However, emerging evidence increasingly supports the involvement of the brain–gut axis in the pathophysiology of IBS-D. In this study, we identified the mPFC-LC pathway as a crucial neural circuit in the pathogenesis of intestinal dysmotility in NMD + RS mice. Specifically, enhanced mPFC glutamatergic projection activities facilitate glutamatergic neuron activation in the LC region, thereby leading to diarrhea (Graphical Abstract). This study provided novel insights into the top-down regulatory mechanisms involved in the onset of IBS-D and hoped to offer a potential new target for clinical non-invasive neural regulation intervention.

An important finding of this study was that enhanced activity of glutamatergic neurons in mPFC played a critical role in driving diarrhea in NMD + RS mice. This finding was supported by a series of experiments. Immunofluorescence staining showed that the c-Fos expression was markedly increased in the mPFC and co-localized with markers for glutamatergic neurons in NMD + RS mice. Furthermore, chemogenetic activation of glutamatergic neurons in mPFC induced diarrhea-like symptoms in CON mice, whereas inhibition of these neurons in NMD + RS mice significantly alleviated the severity of diarrhea. Together, these results confirm that mPFC glutamatergic neurons mediate intestinal dysmotility in IBS-D. The mPFC, as a high-level integrative center for sensory and motor processing, modulates gastrointestinal motility and secretion via descending regulation of the autonomic nervous system [27,28,29,30]. Notably, its superficial cortical location also provided an advantageous site for non-invasive neuromodulation strategies. Techniques such as transcranial magnetic stimulation (TMS) and transcranial direct current stimulation (tDCS) targeted at the PFC, which have been widely used in the treatment of neuropsychiatric disorders, therefore represent promising translational strategies for IBS-D. However, their application requires further clinical validation to address patient heterogeneity in mPFC-LC circuit function. Moreover, standardized stimulation parameters—including site, frequency, intensity, and treatment duration—are currently undefined for IBS-D. Future protocols should be mechanistically informed and tailored to optimize efficacy.

Another key finding of this study is that enhanced activity of glutamatergic neurons in the LC projecting from the mPFC neurons (LC^mPFC → Glu^) mediated diarrhea in NMD + RS mice. Although patients with IBS exhibited increased regional cerebral blood flow and BOLD responses in the LC during visceral stimulation or pain anticipation, the specific role of this LC activation in intestinal motility and its underlying neural circuitry remains elusive. Here, we provided multiple lines of evidence supporting that LC glutamatergic neurons mediate intestinal motility. Firstly, c-Fos expression was significantly increased in the LC of NMD + RS mice and showed high co-localization with glutamatergic neuronal markers. Viral tracing further confirmed direct projections from mPFC neurons to glutamatergic neurons in the LC. Chemogenetic activation of the LC^mPFC → Glu^ neurons induced diarrhea-like symptoms in CON mice, whereas inhibiting these neurons markedly alleviated diarrhea in NMD + RS mice. These results revealed that LC^mPFC → Glu^ neuronal activity regulated intestinal motility in IBS-D. Notably, the high co-expression of tyrosine hydroxylase (TH) in LC glutamatergic neurons suggests their capacity for norepinephrine (NE) co-release. Furthermore, it was reported that high-frequency activity in VGLUT2^+^ terminals may evoke local TH^+^ neurons via microcircuit activation, amplifying NE release and potentially forming a positive feedback looWhile our data underscore a central role for glutamatergic signaling, the specific contribution of LC-derived NE to intestinal motility remains incompletely resolved. We propose that glutamate and NE may regulate intestinal function through distinct spatiotemporal and receptor-specific pathways—for example, NE may act directly on enteric circuits, while glutamatergic projections modulate local LC microcircuits or downstream brain regions. To disentangle the distinct roles of these co-existing neurotransmitters, future studies should employ specific genetic tools (e.g., TH-Cre × VGLUT2-Flp mice) combined with in vivo neurotransmitter monitoring and receptor-specific manipulations to precisely define their functions within the brain–gut axis.

Another notable discovery is that increased activity of glutamatergic neurons in the mPFC activated the LC glutamatergic neurons, thereby mediating diarrhea in NMD + RS mice. Previous studies had shown that the PFC could regulate the neuronal activity in the LC via top-down inputs, thus leading to modulating anxiety, arousal, sensory processing, and cognitive responses [31,32,33]. However, its role in intestinal motility remained unclear. In this study, chemogenetic viral tools were employed to specifically regulate the mPFC–LC circuit and functionally demonstrate its direct involvement in the regulation of intestinal motility. Specifically, activation of mPFC glutamatergic neurons in CON mice induced diarrhea-like symptoms, whereas inhibition of downstream LC glutamatergic neurons alleviated these symptoms. Conversely, in NMD + RS model mice, inhibition of mPFC glutamatergic neurons significantly ameliorated diarrhea-like symptoms, while activation of downstream LC glutamatergic neurons reversed this beneficial effect. Together, these findings revealed that the mPFC–LC circuit is a specific pathway within the brain–gut axis that plays a central role in stress-induced diarrhea in IBS-D. The mPFC and LC are central integration sites for pain and emotional processing [33,34,35]. It means that the mPFC–LC pathway not only offers a neurobiological basis for how psychological stress triggers and exacerbates IBS-D symptoms but may also represent a critical node where anxiety and visceral pain in IBS. It should be noted that this study relied exclusively on chemogenetic approaches. Therefore, future work employing complementary techniques—such as combined optogenetic and chemogenetic modulation—will be valuable for further validating the function of the mPFC–LC circuit.

In conclusion, our study demonstrated the mPFC-LC glutamatergic circuit as a critical pathway in the development of diarrhea in NMD + RS mice, which may provide new mechanistic insights and a potential neuromodulatory target for the clinical management of IBS-D.

## 4. Materials and Methods

### 4.1. Animals

C57BL/6J mice were housed in a specific pathogen-free (SPF) animal facility. The room temperature was maintained at 22–25 °C with a 12 h light/dark cycle. Mice had free access to sterile chow (Suzhou Shuangshi Experimental Animal Stall Food Technology Co., Ltd., Suzhou, China) and drinking water. The conduct of experiments was approved by the Institutional Animal Care and Use Committee of Soochow University [36] (SYXK 2022-0043, Soochow University, Suzhou, China, 15 July 2022 to 14 July 2027).

### 4.2. Establishment of IBS-D Mice Model

The neonatal maternal deprivation combined with restraint stress (NMD + RS) model was employed to induce diarrhea in mice, as described previously [24,25]. Briefly, from postnatal day 2 to day 21, pups in the NMD + RS group underwent 3 h of maternal separation daily. Upon reaching 6 weeks of age, they were subjected to restraint stress. The mice were placed in sterilized centrifuge tubes for 2 h per day over a period of 14 consecutive days, which limited their ability to turn around while allowing limited forward and backward movement [37]. The offspring mice in the CON group received no intervention.

### 4.3. Whole Gastrointestinal (GI) Transit Test

To evaluate gastrointestinal transit time [38,39,40,41,42], mice were fasted for 14 h before the experiment, with free access to water. Subsequently, each mouse was administered 0.2 mL of ink suspension (prepared by mixing ink with a 0.5% sodium carboxymethylcellulose solution), and the time interval from gastric administration to the appearance of the first black fecal pellet was recorded.

### 4.4. Small Intestinal Propulsion Efficiency

To assess small intestinal propulsion efficiency, mice were fasted for 14 h prior to the experiment. Subsequently, each mouse received 0.2 mL of ink suspension through intragastric administration. Twenty minutes later [40], mice were euthanized via cervical dislocation and dissected to reveal the digestive tract. The small-intestinal transit rate was defined as (the distance from the pylorus to the leading edge of the ink/the total length of the small intestine) × 100%.

### 4.5. Colonic Transit Assay

Colonic transit function was determined by the bead expulsion test, as described previously. Mice were fasted for 14 h before the experiment with free access to water. Under isoflurane anesthesia, a smooth plastic tube marked 2 cm from the tip was inserted through the colon [43,44], and a 2 mm-diameter glass bead was advanced 2 cm into the colon. The mouse was placed in a box, and the expulsion of the glass bead was recorded as the colonic transit time.

### 4.6. Fecal Water Content

The fresh feces from each mouse were collected within 2 h in a centrifuge tube to determine the wet weight [38,39,40,41,42,44]. Then the feces were subsequently oven-dried at 60 °C for 12 h to constant weight for dry weight determination. Fecal water percentage was calculated as [(wet weight − dry weight)/wet weight] × 100%. In addition, the amount of feces was recorded as defecation frequency.

### 4.7. Viruses

The virus was microinjected into specific brain regions via a stereotaxic apparatus [45,46]. Briefly, after deep anesthesia in mice, the skull was exposed, and specific brain regions were marked, and then the holes were drilled in the skull using a cranial drill. Viruses were injected into the LC and mPFC of mice at the following stereotaxic coordinates: LC: AP −5.3 mm, ML −0.85 to −0.90 mm, DV −4.3 mm, a volume of virus is 90 nL; mPFC: AP +2.2 mm, ML +0.40 mm, DV −2.8 mm, a volume of virus is 150 nL. The viruses were injected slowly at a rate of 10 nL/min using a microinjection pumAfter injection, mice underwent cardiac perfusion to facilitate viral expression. Viruses used in this study include the following: AAV2/9-VGLUT2-hM3D (Gq)-EGFP, AAV2/9-VGLUT2-hM4D (Gi)-EGFP, AAV2/9-VGLUT2-DIO-hM3D (Gq)-EGFP, AAV2/9-VGLUT2-DIO-hM4D (Gi)-EGFP, AAV2/9-VGLUT2-EGFP, AAV2/R-hSyn-mCherry, AAV2/1-hSyn-Cre, and AAV2/9-EF1α-DIO-EGFP (BrainVTA, Wuhan, China). To activate or inhibit neurons expressing hM3D (Gq) or hM4D (Gi), respectively, clozapine-N-oxide (CNO, Cat# CNO-02, BrainVTA, Wuhan, China) was administered either via intracerebral microinjection or intraperitoneal injection. For central delivery, CNO (0.33 mg/mL) was microinjected (1 μL) into the mPFC or LC through implanted cannulas. For systemic administration, CNO was injected intraperitoneally at a dose of 1 mg/kg.

### 4.8. Immunofluorescence Staining

c-Fos expression in multiple brain regions was measured by immunofluorescence staining [36]. First, frozen brain sections were treated with citrate buffer for 10 min for antigen retrieval and blocked for 1 h. After that, the sections were incubated with primary antibodies at 4 °C overnight. Next, they were washed three times with PBS, each wash lasting 10 min. The sections were then incubated with secondary antibodies for 1 h at room temperature. Finally, after a last wash, they were mounted using DAPI-containing anti-fade medium. Images were captured using an Axio Scope A1 microscope and a confocal microscope (20× immersion lenses, Zeiss, Oberkochen, Germany). For analysis, two non-consecutive sections from each relevant brain region per mouse were used, with each data point representing one brain section. The primary antibodies included anti-c-Fos (1:300, Cat# OB-PGP080, Oasis Biofarm, Zhejiang, China. RRID: AB_2941873), anti-glutamate (1:300, Cat# G6642, Sigma, Livonia, Michigan, USA. RRID: AB_259946), and anti-GABA (1:300, Cat# A2052, Sigma, Livonia, MI, USA. RRID: AB_477652). The secondary antibodies included Donkey anti-Rabbit 568 (1:500, Cat# A-10042, Thermo Fisher Scientific, Waltham, MA, USA. RRID: AB_2534017) and Goat anti-guinea pig 488 (1:300, Cat#GP488, Oasis Biofarm, Zhejiang, China. RRID: AB_3717497).

### 4.9. Data Analysis

All data in this study are expressed as mean ± standard error of the mean (SEM) in this study. Statistical analysis was performed using GraphPad Prism 8.0. Data were tested for normal distribution. Differences between groups were analyzed using an unpaired *t* test, Mann–Whitney U test, or two-way ANOVA followed by Sidak’s multiple comparisons test, as appropriate. A *p*-value below 0.05 was considered statistically significant.

## Data Availability

The original contributions presented in this study are included in the article. Further inquiries can be directed to the corresponding authors.

## Abbreviations

The following abbreviations are used in this manuscript:

IBS-D	Diarrhea-predominant irritable bowel syndrome
LC	locus coeruleus
mPFC	medial prefrontal cortex
NMD + RS	neonatal maternal deprivation plus restraint stress
SPF	specific pathogen-free
BNST	bed nucleus of the stria terminalis
PVN	paraventricular nucleus of the hypothalamus
PVT	paraventricular thalamic nucleus
ACC	anterior cingulate cortex
CNO	clozapine-N-oxide
